# Impact of a combined lower carbohydrate and Mediterranean diet on metabolic syndrome severity: protocol for a randomised controlled trial

**DOI:** 10.1017/S0007114526106357

**Published:** 2026-05-28

**Authors:** Tannia Cyriac, Kate Oetsch, Barbara J. Meyer, Yasmine C. Probst, Lauren A. Roach, Vinicius do Rosario, Monique E. Francois

**Affiliations:** 1 School of Medical, Indigenous and Health Sciences, University of Wollongong, Faculty of Science, Medicine and Health, Australia; 2 University of Wollongong Molecular Horizons Centre for Molecular and Life Science, Australia

**Keywords:** Metabolic syndrome, Diet, Mediterranean diet, Carbohydrate-restricted, Cardiometabolic risk factors

## Abstract

The Mediterranean diet and a low-carbohydrate diet are two popular dietary approaches recommended for cardiovascular and metabolic health, respectively. This trial will compare the combined effect of these diets to either approach alone for the treatment of metabolic syndrome (MetS). Males and females (*n* 222), 30–75 years, with at least three MetS risk factors, will be randomised to one of three diets: (i) traditional Mediterranean (∼55 % of energy carbohydrate:15 % protein:30 % fat), (ii) lower carbohydrate (∼35 % carbohydrate:20 % protein:45 % fat) or (iii) lower carbohydrate Mediterranean (∼35 % carbohydrate:20 % protein:45 % fat) diet for 12 weeks. The primary outcome measure is the MetS severity Z score, a composite score of risk factors, sex and ethnicity. MetS severity Z score will be calculated pre- and post-intervention using fasted blood samples for plasma TAG, HDL-cholesterol and glucose, systolic blood pressure, body weight and waist circumference measures. The findings from this trial will offer new insights into the most effective dietary strategy for managing diabetes and reducing cardiovascular risk in individuals with MetS.

Metabolic syndrome (MetS) is a ‘silent epidemic’ that places increasing pressure on healthcare systems and economies around the world^(1)^. Components of the MetS such as high blood pressure, high blood glucose and high BMI still remain the leading risk factors driving the global burden of disease over the last 2–3 decades^(2)^.

MetS is a cluster of conditions that significantly increases the risk of diabetes, heart disease and stroke^(3,4)^. An individual with MetS has (or is being medicated for) at least three out of five risk factors (central obesity, hypertension, hypertriglyceridaemia, low HDL-cholesterol and elevated fasting glucose)^(5)^. The severity of MetS can be assessed with a continuous MetS severity *Z* score (MetS-Z), a composite score of the MetS risk factors, taking into account sex and ethnicity^(6)^. A higher score is associated with a higher risk of CVD incidence^(6–8)^.

The MetS-associated CVD risk is modifiable with lifestyle behaviours such as dietary modification, which are the first-line treatment, followed by medications when required^(5,9)^. A Mediterranean style of eating, characterised by high consumption of olive oil, fruits, vegetables, whole grains and fish^(10)^, has emerged as a highly recommended dietary pattern for MetS and CVD, endorsed by many organisations including the American Heart Association^(11)^, National Heart Foundation of Australia^(12)^, WHO^(13)^ and healthcare professionals for cardiovascular health^(14,15)^.

Another emerging dietary approach to manage cardiometabolic risk and insulin resistance, obesity and type 2 diabetes (T2D) is the use of lower carbohydrate (LC) diets. The consumption of dietary carbohydrate elevates blood glucose concentrations more than other macronutrients, thereby increasing insulin secretion and placing greater demand on the pancreas^(16,17)^. Consuming a diet that is lower in carbohydrates has been shown to improve insulin sensitivity, glucose metabolism, dyslipidaemia and inflammation^(18–23)^ – conditions often associated with MetS. In recent years, the American Diabetes Association and Diabetes Australia have produced position statements for the use of low-carbohydrate diets in the prevention and treatment of obesity and T2D^(24–26)^.

Combining the Mediterranean diet with an LC approach, that is, reducing carbohydrate intake whilst retaining whole grain foods and increasing *n*-3 fatty acids from fish, nuts and olive oil, was the focus of two earlier studies in people with T2D^(27,28)^. Elhayany *et al*. reported significantly greater reductions in body weight (−10·1 kg), HbA1c (−2 %), plasma TAG (−1·3 mmol/l) and HDL-cholesterol (0·1 mmol/l) following 12 months of a low-carbohydrate Mediterranean approach compared with a low-fat dietary pattern^(27)^. Similarly, Esposito *et al*.^(28)^ reported improved HbA1c (−1·2 %) and plasma HDL-cholesterol (0·10 mmol/l) in people with T2D, and after 4 years of a low-carbohydrate Mediterranean approach, only 44 % of participants required medication compared with 70 % in the low-fat group^(29)^. However, in the time since publication of these studies, there have been no further randomised controlled trials (RCT) to test the effects of the low-carbohydrate Mediterranean diet on different population groups and none in prevalent health conditions such as MetS.

The primary aim of this RCT is, therefore, to investigate the effects of a lower carbohydrate Mediterranean (LCM) dietary pattern compared with two other diets, namely, traditional Mediterranean (TM) and an LC diet, on the MetS-Z in adults with MetS. The hypothesis is that the LCM dietary pattern will have the greatest impact on reducing the MetS-Z score compared with the other two diets. Secondary outcomes include the effects of the dietary approaches on individual CVD risk factors, endothelial function, arterial stiffness, erythrocyte fatty acids, body composition and plasma inflammatory markers.

## Methods

### Study design and overview

A parallel group RCT will be conducted at the University of Wollongong Clinical Trials Research Unit, NSW, Australia, recruiting from Wollongong and the surrounding regions from May 2022 to December 2025. The trial includes online telehealth consultations and in-person data collection at the Clinical Trials and Research Unit, University of Wollongong. Adults with MetS will be randomised to one of three dietary interventions: (i) TM, (ii) LC (∼35 % energy carbohydrate (CHO)) or (iii) LCM (∼35 % energy CHO) dietary pattern for a 12-week period. The primary outcome of the MetS-Z will be assessed for all participants pre- and post-intervention.

### Study integrity

This study will be conducted according to the guidelines in the Declaration of Helsinki, and all procedures involving human participants were approved by the University of Wollongong and Illawarra Shoalhaven Local Health District Human Research Ethics Committee (2021/204). Written informed consent will be obtained from eligible participants prior to data collection by the study co-ordinator. The study was developed as per the CONSORT guidelines and is registered with the Australian New Zealand Clinical Trials registry (ACTRN12622000211763p) at https://www.anzctr.org.au/.

Participants will be randomised in a 1:1:1 allocation, stratified for sex using uneven blocks, to one of the three dietary intervention arms. Allocation will be generated by the statistician, and the Excel file access will be shared with the clinical trials manager in the Clinical Trials Research Unit. The study co-ordinator will generate the alphanumeric code and receive the assigned intervention from the clinical trials manager prior to a participant’s baseline visit.

### Study recruitment

Participants with risk factors of the MetS^(5)^ will be recruited using social media advertising, community notice board posters, the clinical trial recruitment company Trialfacts© and the University of Wollongong Marketing team. Emails will also be sent out to participants registered with the National Diabetes Services Scheme. Interested participants will complete a pre-screening interview over the telephone to determine their eligibility based on the inclusion/exclusion criteria.

Inclusion criteria were age between 30 and 75 years with at least three of the MetS conditions^(5)^ – waist circumference: ≥94 cm in males, ≥80 cm in females; fasting blood glucose: >5·5 mmol/l; HbA1C: ≥6 % and/or oral antidiabetic medications; fasting plasma TAG: ≥1·7 mmol/L; fasting plasma HDL-cholesterol: <1·0 mmol/L in males, <1·3 mmol/L in females; blood pressure: ≥130 mm/Hg systolic or ≥85 mm/Hg diastolic; and non-smoker (>6 months prior to study start) and all medications stable for the previous 3 months prior to trial commencement.

Exclusion criteria included inability to communicate in the English language, any history of CVD, renal or liver disease, current cancer treatment, type 1 diabetes and T2D controlled with insulin or sulfonylureas, pregnant or lactating, any dietary pattern that may impact ability to follow the intervention (vegan/vegetarian/ketogenic), red meat or seafood aversion/allergies and food allergies or intolerances related to all nuts.

### Interventions

All diet interventions follow a duration of 12 weeks. The intervention arms will use the Mifflin–St. Jeor equation to determine individual estimated energy requirements^(30)^. Participants in each arm will receive dietitian-tailored patterns of eating within the below recommendations, depending on the intervention arm they are randomised to. All interventions are modelled^(31)^ with the aim to meet the minimum recommended intakes of the Australian Guide to Healthy Eating^(32)^ and individual recommended daily intakes for micronutrients.

Participants will continue standard care with their primary care provider and all prescription medicines and supplements whilst being enrolled in the study. In consultation with their healthcare provider, participants will be advised to keep their medications unchanged through the intervention period.

#### Traditional Mediterranean diet (TM)

The TM arm is based on the dietary patterns described in the Prevention with Mediterranean Diet study^(10)^ (∼50–60 % of energy CHO; ∼15 % energy protein; ∼25–35 % energy fat) with a focus on olive oil, nuts, seafood, wholegrains, fruit and vegetables. Recommendations for red meat and whole-fat dairy products will be in line with the Australian Guide to Healthy Eating, and red wine (optional) will be in line with the National Health and Medical Research Council Australian alcohol guidelines (2020) of two standard drinks or less per day and at least two alcohol free days per week. This is a deviation from TM diets to align with the Australian alcohol guidelines.

#### Lower carbohydrate Mediterranean diet (LCM)

The combined LC and Mediterranean dietary intervention follows similar guidelines to the TM diet, but with reduced carbohydrates (∼35 % *v*. ∼55 % CHO, i.e. less fruit, breads, cereals whilst retaining whole grains, vegetables) in line with previous LCM dietary studies (∼35 % energy CHO; ∼20 % energy protein; ∼45 % energy fat)^(26,27)^. Recommendations for red meat and whole-fat dairy products will be in line with the Australian Guide to Healthy Eating and red wine (optional) in line with the National Health and Medical Research Council Australian alcohol guidelines (2020) of two standard drinks or less per day and at least two alcohol-free days per week.

#### Lower carbohydrate diet (LC)

The LC dietary intervention will focus on whole foods whilst following an LC dietary pattern including wholegrains, some fruits and vegetables. Whole-fat dairy products, red meats and eggs will be included, whilst olive oil, seafood and nuts will not be encouraged, as a point of difference from the Mediterranean arms. The macronutrient distribution targets will be the same as those of the LCM dietary intervention (∼35 % energy CHO; ∼20 % energy protein; ∼45 % energy fat)^(26,27)^.

Similarities and differences between diets are briefly summarised in Tables 1 and 2.

**Table 1.** Macronutrient composition of intervention diets Table 1 long description.

	Traditional Mediterranean	Lower carbohydrate Mediterranean	Lower carbohydrate
Carbohydrates (%TE)	∼50–60 %	∼35 %	∼35 %
Protein (%TE)	∼15 %	∼20 % (more from white meat, legumes, etc.)	∼20 %
Fat (%TE)	∼25–35 %	∼45 % (more from olive oil, nuts, fish, etc.)	∼45 %

%TE, percent total energy.

**Table 2.** Similarities between intervention diets Table 2 long description.

Traditional Mediterranean	Lower carbohydrate Mediterranean	Lower carbohydrate
	35:20:45	35:20:45
Wholegrains	Wholegrains	Wholegrains
Whole-fat dairy products	Whole-fat dairy products	Whole-fat dairy products
Fish/legumes/nuts	Fish/legumes/nuts	
Olive oil	Olive oil	
≤ 2 standard drinks/d	≤ 2 standard drinks/d	≤ 2 standard drinks/d

### Dietary consultations

A team of 2–3 Accredited Practising Dietitians and a qualified nutritionist will deliver study instructions/protocols and individualised nutrition consults via telehealth. Consults are ∼20–45 min and occur once per week for the first 4 weeks and then once per month for the remainder of the study. Nutrition education topics include the practical aspects of following the allocated dietary pattern, recipe suggestions and instructions for completing a weighed food record. Subsequent consultations address behaviour modification strategies and practical ways to overcome any barriers reported during the consultations. Topics include meal planning and preparation, dining out and how emotions can drive food decisions. The order of education topics will remain the same for all study arms. Each consultation will be documented on a case report form for the dietitian to record the outcomes of the meeting including the provision of any education resources.

To ensure that all topics are consistently covered across the intervention arms, intervention fidelity will be established by discussing the anonymised consultation outcomes during weekly team meetings. This also provides an ongoing quality assurance forum for discussion, knowledge sharing and team problem-solving to help ensure that the advice offered during consultations is equitable.

### Adherence and dietary assessment

Prior to the intervention, a diet history interview will be conducted by an Accredited Practising Dietitian face-to-face or by telehealth with participants to capture habitual dietary pattern information. This assessment will serve as a baseline of habitual intake and will be used to tailor intervention advice as per participants’ dietary preferences.

A 4-d weighed food record (three weekdays and one weekend day) will be requested at weeks 3 and 8 of the trial to determine adherence to the diet intervention. Participants will be informed of the requirement; however, they will not be told in which weeks these assessments will occur to reduce the risk of pre-planning or intentional modification of intake (bias). Weighed food record data will be collected using the *Australian Easy Diet Diary* (Xyris Software (Australia) Pty Ltd) app for iOS and Android smartphones and tablets, and participants will be instructed on its use during the baseline dietary consults. Participants also complete a checklist of weekly food group targets achieved.

The Mediterranean Diet Adherence Screener (MEDAS)^(33)^ will be used at baseline, week 3, week 8 and week 12 to ensure that both Mediterranean diet arms are routinely following the guidelines of the Mediterranean diet. The MEDAS is also completed for participants in the LC diet arm to maintain consistency and monitor that there is an adequate point of difference between interventions. Compliance with the LC interventions (CHO 35 ± 10 % energy) will be assessed using the two weighed food records and weekly checklists/Easy Diet Diary data.

Dietary satisfaction has been linked to long-term diet adherence^(34)^. The Diet Satisfaction-45 survey^(35)^ will be used as a measure of satisfaction pre- and post-intervention. This tool explores changes in dietary satisfaction between the habitual dietary pattern and satisfaction with the randomised dietary arm. In addition, the Theory of Planned Behaviour survey^(36)^, which monitors participants’ attitudes toward the dietary intervention and their willingness to change at different time points, will be completed prior to the intervention diet start.

### Feasibility and safety monitoring

Measures of recruitment and participant retention will be used to assess feasibility. Participants will be encouraged to report any adverse events to researchers throughout the study, which will be escalated to the data safety management committee and reported to the ethics committee. The data safety management committee will assess and advise on the management of the adverse event. The independent data safety management committee (comprised of one clinician, one dietitian and a researcher) will meet half-yearly and as required for study compliance and monitoring for the duration of data collection. The LC arms of the study will be provided with ketone urinalysis strips to detect ketones in the urine. Although we do not anticipate this level of carbohydrate restriction to induce ketosis, participants in these arms will be asked to monitor ketones in urine once per week. If ketones are detected, participants report back to researchers, and carbohydrate adherence will be checked and adjusted, if required, with additional education provided as needed.

Participants will receive their testing visit results on completion of the trial, and any high readings will be flagged for review with their healthcare team.

### Physical activity

Participants will maintain their habitual exercise and physical activities. Physical activity pre- and post-intervention will be assessed with the International Physical Activity Questionnaire – Long Form^(37)^ completed by the participants.

## Outcome measures

**Fig. 1.**
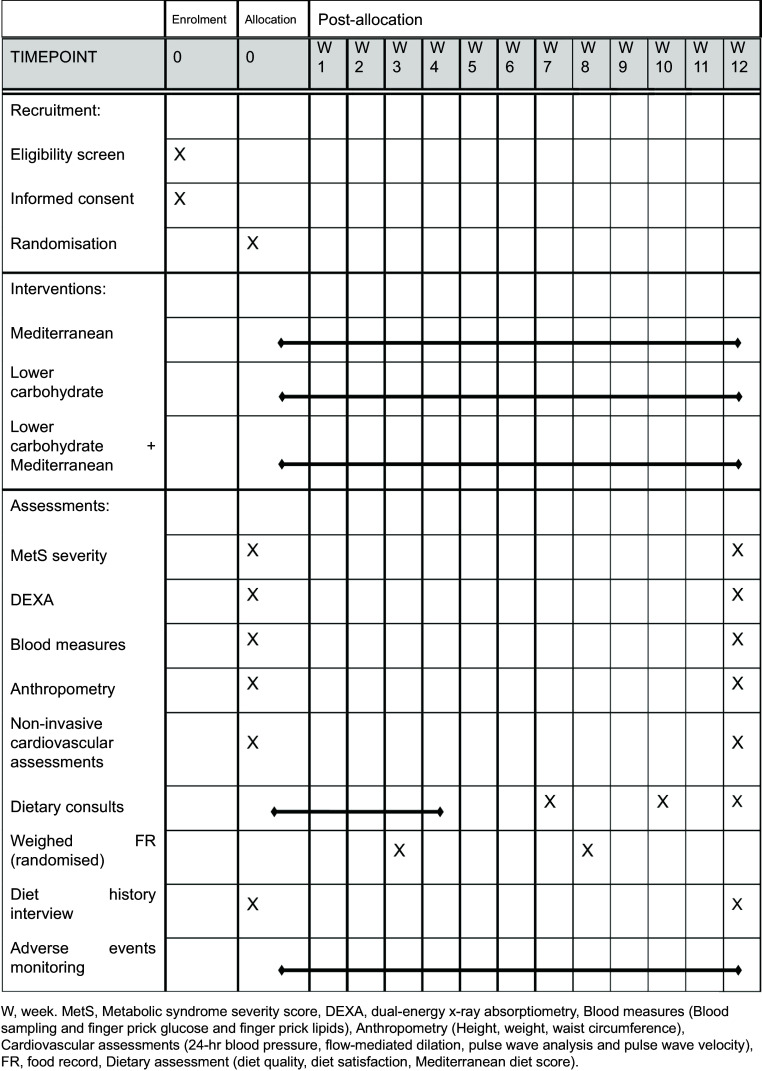
Fig. 1 long description. SPIRIT figure. W, week; MetS, metabolic syndrome; DEXA, dual-energy X-ray absorptiometry; FR, food record.

The study protocol of the outcome measures and timeline is provided in Fig. 1 according to the SPIRIT 2025 statement^(38)^.

### Primary outcome

#### Metabolic syndrome severity Z score

The MetS-Z will be calculated using sex, ethnicity, BMI (height and weight), systolic blood pressure and finger-prick values of fasting plasma HDL-cholesterol, TAG and glucose and is assessed pre- and post-intervention for all participants. The MetS-Z score was developed in 2014 and has since been validated by various studies^(39)^. The score will be calculated pre- and post-intervention using the online calculator available at https://metscalc.org/metscalc/.

Participants will attend the University of Wollongong after a 10–12 h fast to have a capillary blood collection taken for measures of fasting plasma glucose and lipids. Fasting glucose will be measured using a HemoCue glucose analyser (HemoCue Glucose 201 RT glucose; HemoCue AB), and fasting plasma lipids will be measured using a Cobas® b 101 system (Roche Diagnostics). Waist circumference will be measured in duplicate by a researcher using standardised guidelines halfway between the last rib and the iliac crest^(40)^ with an anthropometric tape. Weight will be measured using calibrated body mass scales, without footwear and light clothing, and repeated in the same manner pre- and post- intervention. Resting blood pressure will be measured using an automated blood pressure monitor in the supine position. 24-h blood pressure will also be measured using an Oscar2 ambulatory blood pressure monitor (SphygmoCor interfacing, SunTech Medical), which is worn over a 24-h period and inflates every hour to measure blood pressure and pulse wave analysis.

### Secondary outcome measures

#### Body composition

Body composition will be determined by a whole-body dual-energy X-ray absorptiometry scan using a MEDIX DR, Medilink, narrow fan beam dual-energy X-ray absorptiometry system (InMed). EAZIX software (version V4.2.12.4, DMS Imaging) will be used to assess total and regional fat and fat-free mass. Measures of percentage body fat, visceral adipose tissue and lean body mass will be reported. The dual-energy X-ray absorptiometry system will be operated by a trained member of the research team with a quality control scan run prior to each participant scan.

#### Blood samples

A trained phlebotomist will draw a fasted blood sample from participants, collected in a 10mL EDTA tube. Blood samples will be subjected to centrifugation at 3000 rpm for 10 min at 4°C to separate plasma and erythrocytes, which will be aliquoted and stored at –80°C until samples are analysed in bulk. Plasma samples will be analysed for insulin, inflammatory markers and apo B using commercially available kits. Plasma and erythrocytes will be analysed for fatty acid analysis. In brief, fatty acids from plasma and erythrocytes will be extracted and trans-esterified according to the method of Lepage and Roy^(41)^. Fatty acids will be analysed by flame-ionisation GC (Model GC-17A, Shimadzu) using a 50 mm × 0·25 mm internal diameter capillary column. One microlitre of the sample will be auto-injected into the column, and the individual fatty acids will be quantified using the Shimadzu analysis software (Class-Vp 7.2.1 SP1). Fatty acid peaks will be identified by comparison with known fatty acid standards, and quantification of fatty acids will occur using an internal standard (heneicosanoic acid, Sigma, MO). Fatty acids will be expressed as both the percentage of total fatty acids and the concentration of fatty acids. The Omega-3 Index will be expressed as the sum of EPA and DHA as a percent of total fatty acids^(42)^.

#### Vascular function

Vascular function will be assessed using three domains: endothelial function determined via endothelium-dependent flow-mediated dilation (FMD), arterial stiffness determined by both pulse wave analysis and pulse wave velocity (PWV) and average 24-h ambulatory blood pressure with pulse wave analysis (Oscar 2, SphygmoCor interfacing, SunTech Medical). FMD involves ultrasound imaging (Terason uSmart® 3300) of the brachial artery by a trained researcher. First, participants lay in a rested supine position for 10–15 min, after which a blood pressure cuff is placed on the forearm, positioned 1–2 cm below the olecranon process. An image of a longitudinal section of the brachial artery, located 2–3 cm above the antecubital fossa, is captured using B-mode ultrasound imaging (insonation angle of 60°). A baseline measure of the brachial artery diameter and blood flow is recorded for 1 min, and then the blood pressure cuff is rapidly inflated to ∼60 mmHg above resting systolic blood pressure and held for 5 min. Measures continue until approximately 30 s prior to the cuff being released (ischaemic stimulus) and then for 3 min following the release of the cuff (recovery)^(43,44)^. This recording will then be analysed using Cardiovascular Suite software (Quipu, Italy). The outcome measure of FMD will be reported as (1) absolute change in artery diameter (absolute FMD = postocclusion_mean_ diameter − preocclusion_mean_ diameter) and (2) relative change in artery diameter from baseline [%FMD = 100 × (absolute FMD/preocclusion_mean_ diameter)].

Pulse wave analysis and PWV are measures of arterial stiffness. To measure pulse wave analysis from central blood pressure, a SphygmoCor® XCEL System (AtCor Medical) will be used, and a brachial blood pressure cuff will be inflated to record central aortic pressure waveforms for 5 s. SphygmoCor software automatically analyses these waveforms to derive measures of systolic pressure, diastolic pressure, pulse pressure, aortic pressure, augmentation index and mean arterial pressure. PWV is also measured by the SphygmoCor system; a tonometer is held to the carotid artery for 10–15 s. During this time, the femoral cuff is inflated. Once fully inflated, a 10 s measure of carotid and femoral pressure waveforms is captured. The measure of PWV is determined by the carotid-femoral distance divided by the pulse transit time [PWV (m/s) = (distal – proximal distance)/transit time]^(45)^. PWV measures are performed in duplicate; a third measure is taken if two values differ by > 0·5 m/s.

#### Post-study interview

Following the 12-week intervention and final testing visit, interested participants will be invited to participate in an optional 20–30 min recorded phone interview to discuss their experiences and provide feedback on the study and to help explore barriers and enablers for adherence to the intervention. The structured interview will be conducted over the phone following a structured interview guide (Appendix 1). The interview will be transcribed verbatim by a researcher, with the transcript and audio file stored in the University of Wollongong Cloud storage. Quotes from the interview will be de-identified.

Two researchers will independently code the transcripts. NVivo (QSR International Pty Ltd (2020)) will be used to organise these data. Investigators will use a framework^(46)^ to analyse data, and transcripts will be coded separately. Direct quotes and themes will be indexed to determine the richness of themes with the generation of sub-themes, if required. Lastly, key themes and direct quotes will be tabulated.

### Statistical analysis

186 (62 per group) participants were calculated using a 5 % significance level with a variance and change of 0·2 in the MetS-Z score after a 12-week cardioprotective diet and exercise programme by Hickman *et al*.^(47)^ based on 80 % power with G*Power software^(48)^ for our parallel RCT design. A total of 222 participants will be recruited to account for 20 % drop out. Final data will be analysed using intention-to-treat analyses with a linear mixed model (SPSS, IBM), and covariates (age, weight loss) will be added to the model if required in consultation with a biostatistician. Data will be coded and remain blinded to outcome assessors until all analyses are completed. Statistical significance will be considered as a *p* value < 0·05.

In addition, a per-protocol secondary analysis will be conducted. Compliance with the LC interventions (CHO 35 ± 10 % energy) will be assessed using the two weighed food records and weekly checklists/Easy Diet Diary data. Mediterranean diet adherence will be estimated using average of the MEDAS scores across the study, weekly checklists/Easy Diet Diary data and weighed food records, that is, overall, attaining high consumption of monounsaturated fatty acids; daily consumption of fruits, vegetables, whole grains, fibre, weekly consumption of fish, poultry, nuts and legumes; a relatively low consumption of red meat; and moderate consumption of alcohol^(49)^. MEDAS scores of 8 or above in the Mediterranean arms, which is considered moderate adherence in most studies/trials^(50)^, will be included in the analysis.

## Discussion

The Mediterranean dietary pattern and LC dietary patterns are popular diet approaches for cardiometabolic health. To the authors’ knowledge, this study will be the first of its kind to evaluate two popular dietary approaches, the TM and an LC dietary pattern against a combined LCM dietary pattern for people with the MetS. The study is novel in its approach as previous studies have only compared the LCM to a TM diet and the American Diabetes Association diet^(27)^ or a low-fat diet^(28)^. By comparing all three dietary approaches, this study will ascertain which diet has the greatest benefit in reducing MetS severity (a combination of cardiovascular and diabetes risk factors) and provide an opportunity to begin exploring the dietary factors that may contribute to greater improvements in cardiometabolic health. This is also the first dietary trial to evaluate the change in MetS severity using the MetS-Z score.

The Mediterranean dietary pattern has been shown to be feasible and well adhered to in Australian RCT^(51,52)^, whereas the LC has shown mixed adherence in studies^(22,53,54)^. However, the feasibility of these diets in the free-living general population is unknown. This study will also add to the limited body of literature on the LCM diet by furthering our understanding of its feasibility and acceptability among individuals with MetS within an Australian cohort. Combining these approaches provides a dietary strategy that can be easily adapted and implemented in clinical practice.

Limitations of this study include the self-reported dietary intakes, which are inherently biased. To combat this, a combination of measurement methods including weighed food records, dietary consults, MEDAS and checklists will be used, which, when combined, will provide a measure of both actual and habitual dietary intake to capture less common food sources. Another limitation of this trial is that the diets are not laboratory controlled as foods are not provided to participants throughout, which can lead to poor implementation and adherence to the prescribed diets. Instead, participants are provided with general dietary guidance and will be required to implement this in a free-living setting. This approach was designed to more closely reflect real-world conditions to assess the feasibility of these diet patterns in Australia and to increase the generalisability of the study outcomes. It is hypothesised that the individualised dietary consultations with participants and accompanying resources will help participants achieve dietary patterns that are sustainable. Due to the nature of the dietary advice and resources provided, it will also not be possible to blind participants to their intervention allocation. However, data will be coded and remain blinded to outcome assessors until all analyses are completed.

In summary, the benefits of the Mediterranean diet approach for cardiovascular health are well-established. The evidence-based benefits of an LC eating pattern are emerging, particularly in trials for people who are overweight/obese and/or have T2D. This study will build on prior research and test whether the combined effect of an LCM dietary pattern has greater benefits for MetS-Z and cardiometabolic health in adults with MetS. By evaluating the LCM against the ‘gold standard’^(55)^ TM diet and LC approaches, this study represents a pioneering investigation in Australia aimed at identifying dietary factors that effectively enhance metabolic and cardiometabolic health.

## Enrolment status

The study is currently recruiting participants, with 200/222 participants (as of 2/12/2025) enrolled and 34 dropouts.

## Appendix 1

1. What was your main motivation for deciding to join the Effects of Dietary Approaches on Metabolic Syndrome study?
2. What aspects of your dietary approach did you like or find enjoyable?
3. What aspects of your dietary approach did you dislike or find unenjoyable?
  - Prompt: How did you deal with this?
4. Do you see yourself following this dietary approach now that you have finished the study?
  - If yes, is there anything you would change going forward?
  - If no, prompt why?
5. How well do you think you were able to follow the dietary recommendations provided to you?
6. Could you please comment on the support given to you during the study?
  - Prompt: Enough support? Not enough support? Format? Person? Frequency?
7. How did your family and friends react to you following the dietary approach?
8. How did this diet compare to some you’ve tried in the past, if any?
9. What are some changes to your health, if any, you’ve noticed over the intervention period?
10. What were some other health changes you’ve made influenced by the study during the 12-week period?
11. Is there any other feedback you would like to provide about your experience in the study?

## Fig. 1 Long description

The table is structured with columns for Enrolment, Allocation, and Post-allocation activities across 12 weeks. It includes rows for Recruitment, Interventions, and Assessments. Recruitment activities include Eligibility screen and Informed consent at the enrolment stage, and Randomisation at the allocation stage. Interventions include Mediterranean, Lower carbohydrate, and Lower carbohydrate plus Mediterranean diets, each spanning different weeks. Assessments include MetS severity, DEXA, Blood measures, Anthropometry, Non-invasive cardiovascular assessments, Dietary consults, Weighed food record, Diet interview, and Adverse monitoring, each scheduled at specific weeks. The table provides a clear timeline for each activity, showing when they start and end.

Navigate back to Fig. 1.

## Table 1 Long description

A table comparing the macronutrient composition of different diets. The table has three columns and four rows, including the header row. The columns are labeled Traditional Mediterranean, Lower carbohydrate Mediterranean, and Lower carbohydrate. The rows are labeled Carbohydrates (%TE), Protein (%TE), and Fat (%TE). Row 1: Carbohydrates (%TE), Traditional Mediterranean, ~50-60 percent, Lower carbohydrate Mediterranean, ~35 percent, Lower carbohydrate, ~35 percent. Row 2: Protein (%TE), Traditional Mediterranean, ~15 percent, Lower carbohydrate Mediterranean, ~20 percent (more from white meat, legumes, etc.), Lower carbohydrate, ~20 percent. Row 3: Fat (%TE), Traditional Mediterranean, ~25-35 percent, Lower carbohydrate Mediterranean, ~45 percent (more from olive oil, nuts, fish, etc.), Lower carbohydrate, ~45 percent.

Navigate back to Table 1.

## Table 2 Long description

A table comparing three different diets: Traditional Mediterranean, Lower carbohydrate Mediterranean, and Lower carbohydrate. The table has three columns and five rows, including the header row. The columns are labeled Traditional Mediterranean, Lower carbohydrate Mediterranean, and Lower carbohydrate. The rows include macronutrient ratios, types of foods, and alcohol consumption guidelines. Row 1: Macronutrient ratios: 35:20:45 for both Traditional Mediterranean and Lower carbohydrate Mediterranean, and 35:20:45 for Lower carbohydrate. Row 2: Whole grains are included in all three diets. Row 3: Whole-fat dairy products are included in all three diets. Row 4: Fish, legumes, and nuts are included in all three diets. Row 5: Olive oil is included in all three diets. Row 6: Alcohol consumption is limited to less than or equal to 2 standard drinks per day for all three diets.

Navigate back to Table 2.
